# Air Quality, Pollution Perception, and Residents’ Health: Evidence from China

**DOI:** 10.3390/toxics11070591

**Published:** 2023-07-07

**Authors:** Jie Zhu, Chuntian Lu

**Affiliations:** 1 School of Humanities and Social Science, Xi’an Jiaotong University, Xi’an 710049, China; zhujie1479@stu.xjtu.edu.cn; 2 School of Marxism, Wuxi Institute of Technology, Wuxi 214121, China

**Keywords:** health, air pollution, pollution perception

## Abstract

Environmental and resident health issues associated with air pollution are an area of growing concern for both policy officials and the general public. In recent years, China has been accelerating the construction of a Beautiful China and a Healthy China, with the aim of protecting and improving the environment and ensuring public health. In this study, we aimed to explore the impact of air quality and air pollution perception on residents’ health. This study used the 2017 Chinese General Social Survey data to measure self-rated health, mental health, and air pollution perception. Using matched socioeconomic indicators and air pollution data, we analyzed the relationship between subjective perception of air pollution, objective air pollution data, and residents’ health. The results showed the following: (1) Air pollution perception has a significant negative impact on self-rated health and mental health. Thus, it needs more consideration to reduce environmental health risks. (2) Objective air pollution has a significant negative impact on mental health. At the same time, its effect on self-rated health was insignificant. These results provide empirical evidence supporting the Chinese government’s decision to invest more in combating air pollution and ensuring the health of Chinese residents.

## 1. Introduction

As a critical indicator of environmental quality, air is a natural resource that humans cannot live without. Therefore, air pollution is one of the primary environmental risks that affects human health. According to the World Health Organization’s “World Health Statistics Report 2021”, air pollution, as an environmental risk factor, is associated with various acute and chronic diseases, such as cardiovascular disease, stroke, respiratory disease, and cancer; air pollution caused approximately 7 million deaths globally in 2016 [1]. Since the implementation of its reform and opening-up policy, China has experienced rapid economic development, which significantly improved the living standards of its people. However, the resulting extensive economic development has led to persistent environmental pollution problems, particularly severe air pollution. The health risks and hazards caused by air pollution have become a public concern. China’s ecological civilization construction emphasizes solving prominent environmental problems that harm public health, and the National 14th Five-Year Plan and 2035 Vision Goal emphasize the establishment of a public health impact assessment system. Therefore, revealing the impact of air pollution on residents’ health in China is of practical significance for assessing the health risks of air pollution and promoting healthy living environments.

In recent years, research on public health and air pollution has received increasing attention. Firstly, the health risks associated with air pollution are gradually increasing. According to a global burden of disease study published in *The Lancet* in 2017, China is one of the countries that is most severely affected by air pollution, with 1.1 million deaths caused by atmospheric PM_2.5_ pollution each year, indicating an increase of 17.5% compared to 1990 [2]. A Chinese burden of disease study published in *The Lancet* in 2019 showed that air pollution is the fourth largest health risk factor in China [3]. Secondly, air pollution has effects on both physical and mental health. In terms of the impact on physical health, existing medical evidence indicates that both short-term and long-term exposure to air pollution can lead to acute and chronic health issues [4]. In terms of mental health, some studies have shown that air pollution factors, including NO_2_, SO_2_, PM_10_, and PM_2.5_ concentrations, have significant negative effects on people’s happiness and mental health [5,6,7,8,9,10] and can also increase the incidence of depressive symptoms [11]. Recently, some studies have reported that indicators of self-reported health status, such as disease severity and depressive symptoms, are associated with perceived air pollution rather than measured air pollution [12]. Scholars have conducted extensive research on the relationship between air pollution and health. However, there are still some shortcomings and areas for further research. Firstly, scholars have mostly studied the health effects of air pollution from a physiological perspective; even though there has been discussion around mental health in recent years, it is still relatively limited. Additionally, the effects of air pollution on physical and mental health are generally discussed separately. Overall, research on the health effects of air pollution is not yet systematic and comprehensive. Secondly, current research mainly focuses on developed countries [13], and there is less research on developing countries. Influenced by factors such as differences in air quality standards, the explanatory power of research results in developing countries is still limited [14]. China is promoting the construction of a healthy and beautiful China, hoping to achieve the goal of safeguarding public health as well as protecting and improving the environment. This requires more academic attention. Thirdly, the health assessment field related to air pollution perception should be optimized. In recent years, with the rise of risk perception research, air pollution as an environmental risk has also attracted some scholars’ attention [15,16]. Currently, only a few studies have applied air pollution perception to health impact assessments, and more in-depth empirical research is needed.

Deguen et al. [17] proposed that a comprehensive assessment of the health effects of air pollution must include both physiological and mental impacts. This study measured residents’ health status from two aspects: self-rated health and mental health. Existing research has shown that self-rated health can comprehensively reflect individual health status, and scholars generally use self-rated health as a proxy indicator of physical health status [18,19,20,21,22,23,24]. This study used regression models to explain self-rated health and mental health in terms of the public’s subjective perception of air pollution, objective air pollution data, and social and economic variables. We mainly used data from the 2017 Chinese General Social Survey (CGSS2017), as well as provincial economic and environmental indicators. The results showed that the higher the air pollution perception, the worse the self-rated health and mental health. Additionally, the higher the objective air pollution, the worse the mental health. However, objective air pollution does not directly affect self-rated health. These findings will be of significance for research and policies to improve air quality and public health.

This study’s main contributions are as follows: First, we evaluated the health effects of air pollution from the perspectives of self-rated health and mental health, taking into account the possibility of different impacts between objective air pollution and air pollution perception. The inclusion of both research perspectives is novel and comprehensive. It expands on previous studies and more closely links air pollution perception and residents’ health, thus enriching the application of air pollution perception and contributing to a deeper understanding of environmental health risks. Second, the current empirical literature on the perception of air pollution by Chinese people is mainly based on small survey samples of specific residents or extensive social surveys conducted nearly a decade ago. This paper provides evidence based on reliable data and a recent representative social survey of China. The results provide empirical evidence supporting the Chinese government’s decision to invest more in combating air pollution and ensuring the health of Chinese residents.

This paper is structured as follows: Section 2 presents a review of the literature focusing on the health risks of air pollution, pollution perception, and its health effects and puts forward the research hypothesis. Section 3 describes the source of the survey data, the design of the variables, and the methods. Section 4 introduces and analyzes the concrete empirical results. Finally, Section 5 and Section 6 provide the discussions and conclusions.

## 2. Literature Review

### 2.1. Health Risks of Air Pollution

Early research on the health effects of environmental pollution was based on the health production function theory pioneered by Grossman [25]. Subsequently, Gerking et al. [26] incorporated environmental factors into the health production function and examined the impact of environmental factors on health depreciation rates. Existing research has mostly analyzed the effects of pollution on residents’ health from an epidemiological perspective, with pollution mainly causing respiratory, cardiovascular, and heart diseases [27,28]. Air pollution is an important source of environmental pollution, and the health effects of air pollution have been studied and analyzed by scholars in the fields of epidemiology, health economics, sociology, and other related fields.

Many scholars have studied the health risks associated with air pollution by examining its relationship with mortality rates and various diseases. Francesca et al. [29] analyzed the relationship between air pollution and mortality rates in 88 of the largest cities in the US from 1987 to 1994 and found that, in most areas, the previous day’s PM_10_ concentration was positively correlated with total mortality rate. Chen et al. [30] systematically evaluated the health effects of air pollution in China using a regression discontinuity design based on data collected from 1981 to 2000. Their results showed that a 100 μg/m^3^ increase in Total Suspended Particulate (TSP) concentration in a long-term living environment was associated with a 14% increase in mortality rate and a 3-year reduction in life expectancy. The increase in the mortality rate was mainly due to an increase in cardiovascular and respiratory diseases. Regarding the relationship between air pollution and infant mortality, Chay [31] found that reducing air pollution levels could reduce the number of infant deaths. Currie et al. [32] reached a similar conclusion in their study.

Regarding the relationship between air pollution and health, most of the research studies have focused on the effects of air pollution on physical health. However, in recent years, some studies have begun to explore how air pollution affects people’s mental health. Compared to physical illnesses, which have a certain latency and lag, intense and negative psychological experiences are more direct and prominent. In this sense, mental health is an issue that economists, sociologists, and psychologists can and should explore in order to prevent and reduce the harm of environmental problems on mental health.

In their studies on air pollution and subjective well-being, Marques et al. [33] used a quasi-experimental design to compare the subjective well-being of residents living in industrial and non-industrial areas. They found that residents living in highly polluted industrial areas not only had lower subjective well-being but also higher levels of anxiety and depression. This was because the air pollution affected individuals’ perception of the risk of illness. Even if they were not ill, individuals exposed to poor air quality for a long time would have increased worries and fears about getting sick, leading to a decrease in their subjective well-being and even anxiety and depression. Some scholars have conducted research on the relationship between air pollution and anxiety and psychological stress [34,35]. Regarding air pollution and depressive symptoms, Block et al. [36] pointed out that air pollution could cause systemic inflammation, cerebrovascular damage, and neurodegenerative changes, thus increasing the risk of depression. Many scholars have empirically analyzed the relationship between the two, and most studies have shown a positive correlation between air pollution and depression. Lim et al. [37] used survey data from Seoul, South Korea, and found that air pollution increased the relative risk of depression in the elderly. Pun et al. [38] used data from the National Social Life, Health, and Aging Project (NSHAP) in the US and studied the relationship between air pollution and depressive and anxiety symptoms in older people. Their results showed that PM_2.5_ was significantly positively correlated with depressive and anxiety symptoms in older people, showing a stronger correlation among individuals with lower socioeconomic status and related chronic diseases. However, some studies have found no positive correlation between air pollution and depressive symptoms [39].

In summary, air pollution has negative effects on human health, with research mainly focusing on the physiological aspects and primarily targeting developed countries. There has been relatively little research on the relationship between air pollution and mental health, especially in developing countries. Recently, foreign scholars have begun to pay attention to this relationship. In China, research on the health effects of air pollution is relatively scarce, and descriptive statistical analyses are more common than empirical analyses. Therefore, it is necessary to use China’s empirical data to analyze the health effects of air pollution. This study used self-rated health as a measure of physiological health, which is a commonly used measurement method in studies on residents’ health. Therefore, we proposed the following hypotheses:

**Hypothesis 1** **(H1):** *The higher the objective air pollution level, the worse the self-rated health will be.*

**Hypothesis 2** **(H2):** *The higher the objective air pollution level, the worse the mental health will be.*

### 2.2. Perception of Pollution and Its Health Effects

There has been a significant amount of research on environmental perception, which includes evaluations of the overall environment as well as specific elements, such as water and air quality [40,41]. Perception of environmental pollution refers to an individual’s perception of pollution in their environment. Previous studies have focused on the impact of pollution levels, resident characteristics, and social environments on the perception of environmental pollution. Most studies suggest a positive correlation between pollution perception intensity and pollution levels [42,43]. However, some scholars argue that pollution perception is mainly influenced by individual characteristics rather than the actual pollution levels of their environment [44]. In the field of health effects research, most scholars have not explicitly used the concept of environmental pollution perception and have not distinguished between objective environmental pollution and subjective environmental pollution. However, distinguishing between subjective and objective environmental pollution is important, particularly when examining subjective evaluation indicators, such as happiness, life satisfaction, and health perception. Subjective environmental pollution is a more intuitive reflection of the relationship between environmental pollution and individual perception [45]. Scholars have typically focused on the impact of air pollutants (such as SO_2_, NO_2_, PM_2.5_, or PM_10_) on individual health from an objective environmental pollution perspective. However, individuals have different levels of sensitivity to environmental pollution, and subjective indicators are more intuitive in reflecting the relationship between environmental pollution and individual health perception.

The attention from the fields of sociology and psychology to air pollution extends beyond its psychological effects to include public perceptions of air pollution. Focusing on the subjective judgments and understanding of the general public regarding air pollution (such as their knowledge of its causes, severity, and harms), surveys or attitude polls are used to understand public attitudes (in contrast to objective data or expert opinions), providing powerful references for the formulation and implementation of public policies related to air pollution. Since the mid-twentieth century, researchers have conducted a large number of surveys and studies on public perceptions of air pollution, achieving fruitful research results [15,46]. There is no consensus on which factors are involved in the public’s perception of air pollution in the existing research. Although the existing research lacks dialogue, the conclusions drawn from different studies consistently show that the perception of air pollution is a subjective construction process or result that does not depend entirely on objective air pollution status. On the one hand, public perception of air pollution itself is a subjective judgment based on life experience and intuitive feelings and may, therefore, be inconsistent with objective data or information. Although most people acknowledge the scientific and necessary nature of objective data, the public’s judgment of air quality is mainly based on sensory experiences, such as vision and smell, rather than air quality monitoring data, and there is no significant correlation between the two. Especially when they are inconsistent or conflicting, the public usually refuses objective data and insists on subjective judgment [47]. At the same time, public perception of air pollution has important practical significance: public exposure to air pollution is largely determined by their judgment of air pollution quality, and public attribution of air pollution may also affect their response to relevant public policies; thus, public perception is also one of the evaluation criteria to determine the effectiveness of air pollution control. On the other hand, the impact of objective information on public perception of air pollution is limited. Even with a large amount of available air pollution information, the public’s attention to and recognition of this information is not high [48]. Beaumont et al. [49] further pointed out that the public is not a homogeneous group, and differences in age, gender, social class, culture, and economic status may affect the effectiveness of risk communication strategies, and attention should be paid to the usefulness of relevant information for different groups. In Bush et al.’s [50] view, the public does not just passively receive air quality information in a social vacuum but reflects on and evaluates air pollution information based on their social and cultural backgrounds, local knowledge, and cognitive processes. Furthermore, subjective air quality is a subjective construction that may be influenced by many factors, such as education level, gender, personality traits, and socioeconomic status [51,52,53].

Previous research has shown that the impact of air pollution on psychological effects largely depends on perceived levels of pollution rather than objective levels of pollution [54]. Liao et al. [45] specifically compared the effects of subjective and objective air quality on life satisfaction. Clearly, the above studies illustrate that there are complex and rich psychological mechanisms underlying the relationship between air pollution and health, and this study focused on the perception of pollution. Therefore, we proposed the following hypotheses:

**Hypothesis 3** **(H3):** *The higher the air pollution perception, the worse the self-rated health will be.*

**Hypothesis 4** **(H4):** *The higher the air pollution perception, the worse the mental health will be.*

### 2.3. Other Factors

Health is related to demographic characteristics, such as age, gender, marital status, education, and socioeconomic status [55,56]. Wilkinson [57] proposed that people determine their relative position by comparing themselves to others around them, and a lower relative position can make people feel disadvantaged, which can lead to health problems, such as cardiovascular disease and depression. Since the 1970s, socioeconomic status has received increasing attention in research on resident health. There is not only a significant correlation between socioeconomic status and resident health, but socioeconomic status differences are also one of the important causes of health inequality. Socioeconomic status is a concept with complex connotations, which is usually measured by indicators such as income, years of education, and occupational status; a socioeconomic status index can also be constructed based on differences in occupational status [58]. A study on the health of residents in 22 European countries showed a robust and sustained relationship between socioeconomic status and resident health [59]. The same conclusion has also been verified in China [60,61]. Based on existing research, the impact of socioeconomic status differences on resident health advantages mainly comes from the following aspects: an increase in income can improve personal living environments and increase the ability to obtain medical resources; a good educational background makes it more advantageous in obtaining medical knowledge and economic and social resources [62]; and upward mobility in occupational status is often accompanied by improvements in work environment and physical activity, which reduce health risks [63]. In the literature on environmental health risks, some studies have further examined the issue of health inequality caused by environmental pollution, and the main view is that groups with low socioeconomic status bear higher health risks due to their greater exposure to environmental pollution [64]. It is not difficult to see that environmental pollution is still an important transmission mechanism for affecting health inequality. Due to the different abilities of individuals with different socioeconomic status to avoid environmental risks, the differentiated exposure level of environmental pollution becomes a source of health and social inequality. This also means that increasing the intensity of environmental regulation is one of the important breakthroughs to reduce pollution exposure risks and achieve environmental health equity.

Although air pollution has negative impacts on health, the severity of these impacts is influenced by the social vulnerability of specific groups [65,66]. The interplay between air pollution and social factors in affecting health has led scholars to explore the “triple jeopardy” of health, social, and environmental inequalities [67]. Research on the differential health impacts of air pollution exposure and environmental justice is a popular topic in health geography [68]. Scholars are concerned with issues of environmental justice and health inequalities arising from the uneven distribution of products, services, pollution exposure, and other factors related to social and economic development [69]. Studies on the relationship between PM_2.5_ and mortality risk have focused on vulnerable groups, such as infants, pregnant women, and the elderly [70,71]. The impact of PM_2.5_ on population health in China varies significantly between urban and rural areas [72]. Air pollution has a more severe impact on the health of vulnerable groups with a lower socioeconomic status, such as those with low income and education levels with poor working conditions and living in environments with high indoor pollution levels [73]. When environmental pollution interacts with health, socioeconomic factors, and inequality issues, it can lead to an environmental health poverty trap, resulting in a vicious cycle of pollution exposure and health hazards [74]. Socioeconomic factors can modulate the relationship between air pollution and health. We focused on the impact of the level of economic development. Urbanization in China has led to more complete medical and health resources, providing opportunities for improving residents’ health and mitigating the impact of air pollution on health [75,76].

## 3. Materials and Methods

### 3.1. Data

#### 3.1.1. Individual-Level Data

The data used in this study were mainly from the CGSS2017. The Chinese General Social Survey, which was initiated in 2003 by the Renmin University of China and the Hong Kong University of Science and Technology, is a nationwide, comprehensive, and continuous academic survey project that has become an important source of micro-econometric research data in China. The CGSS2017 was conducted in 2017, using a multi-stage stratified sampling procedure and with a population coverage of adults. The survey was conducted through face-to-face interviews, and the collected data and materials were cleaned, processed, archived, standardized, and internationalized according to international standards. The survey data were released in 2020. For the purposes of this study, a total of 4132 valid samples were processed, which were collected from 28 province-level administrative regions, including Beijing, Shanghai, Heilongjiang, Henan, Shaanxi, and Guangdong. The mean or median values were used to fill the missing values of variables in some samples.

#### 3.1.2. Provincial Hierarchical Data

This study also used province-level data, specifically the socioeconomic and environmental indicators of 28 provinces in 2016 where the respondents were located. Socioeconomic data were taken from the China Statistical Yearbook and the China Statistical Report on Internet Development, which was published by the China Internet Network Information Center. Air pollution data were sourced from the China Statistical Yearbook, which contains information on the main pollutants emitted from waste gas in different regions. Since the general population is directly exposed to the air environment, the degree to which residents’ health is affected by air pollution is greater than that of other environmental factors. This can compensate for any potential deficiencies in matching macro-level data with micro-level individual data and, to some extent, alleviate endogeneity issues [77]. To accurately evaluate the impact of pollution on residents’ health, the collection of macro-level data and survey sample data was not strictly synchronized in time. Therefore, this study further addressed the endogeneity issues by lagging the environmental indicators [78]. On the one hand, the impact of air pollution on residents’ health may take some time to manifest, which often interferes with accurate assessments of this long-term effect. On the other hand, it is impossible for air pollution indicators that occur earlier to be affected by the subsequent health of residents. Lagging the environmental indicators can also eliminate the possible bidirectional causal relationship between air pollution and residents’ health. However, the macro-level data used in this study also have certain limitations. We could only locate respondents to their respective provinces, although the city or county level is undoubtedly more accurate than the provincial level. However, due to the limitations of the CGSS2017 data, the respondents could not be located to their respective districts and counties, so we could only collect macro-level data at the provincial level.

### 3.2. Variables

#### 3.2.1. Dependent Variable

This study measured residents’ health from two aspects: self-rated health and mental health. Compared to a single measurement indicator, self-rated health is a subjective evaluation of one’s own health status after considering various complex factors, such as disease severity, family history, and health stability. This measurement method conforms to the sufficiency of psychometrics and the reliability and validity of statistics. Therefore, despite certain deviations from objective health, it is still considered an effective predictive indicator of mortality and other functional limitations in many countries and regions. In the CGSS2017, the specific question about self-rated health was, “How do you rate your current physical health?” with a total of seven possible answers. The ranking was as follows: 1 meant “very unhealthy”, 2 meant “somewhat unhealthy”, 3 meant “average”, 4 meant “somewhat healthy”, 5 meant “very healthy”, 98 meant “don’t know”, and 99 meant “refuse to answer”. For mental health, the specific question in the CGSS2017 was, “How often have you felt depressed or down in the past four weeks?” with a total of seven possible answers. These answers were as follows: 1 meant “always”, 2 meant “often”, 3 meant “sometimes”, 4 meant “rarely”, 5 meant “never”, 98 meant “don’t know”, and 99 meant “refuse to answer”. We converted ordinal variables into interval variables, which is a widely used method, and the results did not change significantly.

#### 3.2.2. Independent Variables

For objective indicators of air pollution, we selected the SO_2_ index and matched the per capita SO_2_ emissions in the provinces in which the respondents lived. Compared to other environmental pollutants, air pollution is more mobile, has a wider impact, and does not discriminate against different populations, making its negative externalities more apparent. Common air pollutants include SO_2_, NO_2_, CO, O_3_, and smoke and dust particles. Due to China’s heavy reliance on coal and other resources for industrial production activities, industries such as power generation, metallurgy, and chemical engineering, emit large amounts of SO_2_ and smoke and dust particles, which is the main reason for the recent surge in PM_2.5_ levels in China. Air pollution mainly affects mental health by causing physical discomfort, triggering inflammatory reactions, and exacerbating emotions such as anxiety and depression. The medical literature has also found that SO_2_ in the air is one of the main causes of respiratory-related diseases caused by air pollution [79]. In addition, in the robustness test section, this study further introduced per capita smoke and dust particle emissions at the provincial level as an air pollution indicator in order to comprehensively investigate the impact of air pollution on health. Atmospheric PM mainly comes from direct emissions of smoke and dust particles from pollution sources, including TSP, PM_10_, and PM_2.5_; PM_2.5_ itself contains various harmful substances to the human body [80], so smoke and dust particles can comprehensively characterize air pollution conditions. The results show that the core conclusions of this study are not sensitive to the selection of air pollution indicators.

For a subjective indicator of air pollution, we chose the air pollution perception. This variable represents an individual’s subjective evaluation of their surrounding air environment. Subjective pollution indicators represent the sensitivity of different individuals to air pollution, and subjective pollution perception variables can more intuitively reflect the relationship between pollution and health. In the CGSS2017, the corresponding pollution question was about an individual’s satisfaction with their surrounding environment. The lower an individual’s satisfaction with their surrounding environment, the greater the degree of pollution in their surrounding environment, making it a characteristic variable. Therefore, in this study, we represented the degree of pollution perception from light to heavy based on environmental satisfaction. We reverse coded the answers, so that higher values represent a more severe subjective pollution level.

#### 3.2.3. Control Variables

Based on Grossman’s health demand theory, this study selected a rich set of indicators as control variables at both the individual and provincial levels to mitigate estimation bias caused by omitted variables. Following the approach of Akpalu et al. [63], individual-level control variables included the socioeconomic status and health-needs characteristics of residents. Specifically, socioeconomic status is a comprehensive measure of an individual’s relative social and economic status compared to others based on factors, such as education, marriage, hukou (household registration), and income, from the fields of sociology and economics. In China, hukou refers to a record of the identity, place of residence, family relationships, and changes of each citizen implemented by the household registration authority according to law. Since the establishment of the system of separate management of agricultural and non-agricultural populations in 1955, the academic community has attached great importance to its social effects [81]. The socioeconomic status characteristics in this study mainly included gender, age, education, income, hukou, political status, self-assessed class, marriage, family size, and region. The educational variable adopted a linear measurement method to measure the total number of years of school education. The income data were derived from the questionnaire about annual income in 2016, and this study used the logarithmic individual annual income to measure it. Gender, hukou, political status, and marriage variables were dummy variables, with female = 1 and male = 0; urban = 1 and rural = 0; a member of the Communist Party = 1 and other = 0; and married = 1 and unmarried = 0. Self-assessed class is an ordinal variable. Region was classified according to the classification standards of the National Bureau of Statistics of China, where the western region = 1, the central region = 2, and the eastern region = 3, with a larger number indicating a greater regional advantage. Health needs are an important factor affecting residents’ self-reported health status, and due to the limitations of the questions in the CGSS2017, the health-needs characteristics in this study mainly focused on daily activity ability. The specific question in the CGSS2017 was, “In the past four weeks, how often has your health affected your work or other daily activities”. There were seven answer options: 1 meant “always”, 2 meant “often”, 3 meant “sometimes”, 4 meant “rarely”, 5 meant “never”, 98 meant “do not know”, and 99 meant “refuse to answer”.

For the social and economic development of the respondents’ provinces, we used three indicators, namely the per capita GDP (in RMB 10,000), the GDP growth rate, and the internet penetration rate of each province.

Table 1 reports the descriptive statistics of the main variables used in our regression analysis from the CGSS2017, as well as air pollution and socioeconomic indicators for 2016. Obs represents the number of observations, M represents the mean, and SD represents the standard deviation.

### 3.3. Methods

With the help of STATA14.0 software (StataCorp LLC, College Station, TX, USA), this study explored the influencing factor models of residents’ self-rated health and mental health from the aspects of individual socioeconomic and demographic variables, health-needs characteristics, macroeconomic development, air pollution perception, and the air pollution levels of their provinces through regression analysis. For the regression analysis, we used the following steps: Firstly, the null model, also known as the one-way analysis of variance model, means there are no explanatory variables at the individual and regional levels. This can be expressed mathematically as:

(1) $$\mathrm{Level}\;1:\;Y_{ij}=\beta_{0j}+r_{ij}$$

(2) $$\mathrm{Level}\;2:\;\beta_{0j}=\gamma_{00}+u_{0j}$$

where $Y_{ij}$ is the score of member *i* on self-rated health or mental health in province *j*, $\beta_{0j}$ is the average score of self-rated health or mental health in province *j*, $r_{ij}$ is the residual, $\gamma_{00}$ represents the grand mean of the outcome, and $u_{0j}$ is the random effect associated with province *j*. The purpose of this model is to decompose the total variance at the individual and regional levels, but it can also calculate the intra-class correlation coefficient (ICC). A high ICC indicates that there is more variation between groups, while a low ICC indicates that the total variance can be explained by within-group variation.

If there were significant differences in residents’ evaluation scores among the provinces, we used a multilevel model. This paper used an improved multilevel model, namely the random intercept model. The level two model incorporates a high-level explanatory variable $W_{j}$, which is solely used to predict $\beta_{0j}$, while explanatory variables are also included in level one. This can be expressed mathematically as:

(3) $$\mathrm{Level}\;1:\;Y_{ij}=\beta_{0j}+{\beta_{1j}X_{ij}+r}_{ij}$$

(4) $$\mathrm{Level}\;2:\;\beta_{0j}=\gamma_{00}+{\gamma_{01}W_{j}+u}_{0j}$$

If there were no significant differences, we used a multiple linear regression model. The multiple linear regression model is a linear regression model that targets one dependent variable (Y) and two or more independent variables ($X_{1},X_{2},\ldots ,X_{k}$). This can be expressed mathematically as:

(5) $$Y=\beta_{0}+\beta_{1}X_{1}+\beta_{2}X_{2}+\ldots +\beta_{k}X_{k}+\varepsilon$$

The independent variables $X_{1},X_{2},\ldots ,X_{k}$ jointly affect a dependent variable Y. The constant term $\beta_{0}$ represents the intercept of the regression line; $\beta_{1},\beta_{2},\ldots ,\beta_{k}$ represent the slopes corresponding to each independent variable; and ε is the error term. These models are standard models, so they are not explained in detail in this paper.

In the regression analysis, we test hypotheses about regression coefficients. A research hypothesis is a hypothesis that is hoped to be supported during the research process. When inferring about a population using a random sample, we do not directly test the research hypothesis but indirectly obtain the possibility of the research hypothesis being correct by testing the opposite hypothesis, which is called the null hypothesis (H_0_). In the research process, the null hypothesis is the hypothesis that the researcher hopes to be rejected. That is to say, the possibility of the null hypothesis being correct is very small, indirectly affirming the research hypothesis. The *p*-value is the probability of observing the sample results in the sampling distribution if the null hypothesis is correct. As the *p*-value decreases, the conclusion becomes more reliable.

## 4. Results

### 4.1. Baseline Regression Analysis

Table 2 reports the setting and regression analysis results of the self-rated health model. Model 1 shows a two-tier, completely unconditional model, from which we can determine how much of the total variance is explained by the individual- and region-level variables. The within-group standard deviation was 1.132, and the between-group standard deviation was 0.075. By calculating the ICC, we found that 6.2% of the variance in self-rated health scores was due to differences across provinces. Following the empirical rule of determining whether to adopt a stratified model based on whether the ICC value is greater than 0.059 [82], this study used the multilevel model for self-rated health.

The Model 2 results show that age (β = −0.01, *p* < 0.001), education (β = 0.02, *p* < 0.001), income (β = 0.01, *p* < 0.05), hukou (β = 0.09, *p* < 0.01), self-assessed class (β = 0.08, *p* < 0.001), and daily activity ability (β = 0.48, *p* < 0.001) were significantly related to self-rated health. However, there was no significant correlation between province-level per capita SO_2_ emissions (β = 2.54, *p* > 0.05) and self-rated health; thus, Hypothesis 1 is not supported. This is similar to the study by Orru et al. [54], which showed that the level of air pollution exposure did not significantly influence health risk perception, symptoms, or diseases. Furthermore, age was negatively correlated with health, meaning that older people tended to have worse self-rated health. People with higher education levels tended to have better self-rated health. Personal income had a positive impact on self-rated health. A possible explanation is that a higher income level can provide people with better living environments and medical services, which leads to better health. People with an urban hukou tended to have higher self-rated health than those with a rural hukou. In addition, the higher the self-assessed social class, the better the self-rated health was, and the stronger the daily activity ability, the better the self-rated health was.

In Model 3, air pollution perception (β = −0.04, *p* < 0.001) was added. The more severe the air pollution that residents perceive was, the worse their self-rated health was, thereby supporting Hypothesis 3.

Table 3 presents the setup and regression analysis results of the mental health model. Model 5 is a two-tier, completely unconditional model. The within-group standard deviation was 0.943, which is much larger than the between-group standard deviation (0.048). The ICC value was 0.049, which means that 4.9% of the variance in mental health evaluation scores is explained by the differences between the provinces. Therefore, for mental health, this study used multiple linear regression instead of the multilevel model.

The Model 6 results show that age (β = 0.01, *p* < 0.001), education (β = 0.01, *p* < 0.01), self-assessed class (β = 0.06, *p* < 0.001), marital status (β = 0.08, *p* < 0.05), region (β = 0.07, *p* < 0.05), daily activity ability (β = 0.38, *p* < 0.001), and per capita SO_2_ emissions (β = −8.17, *p* < 0.01) were significantly correlated with mental health. The higher the per capita SO_2_ emissions, the worse the mental health of residents was. Therefore, Hypothesis 2 is supported by the data. In addition, older people had better mental health. This study’s findings are consistent with Zhang et al.’s [11] findings. A possible reason is that older people have a longer life span and richer life experiences, which make them more adaptable to living in an environment with poorer air quality. People with higher education levels had better mental health. The higher the self-assessed social class, the better the mental health was. Married people had better mental health. The stronger the daily activity ability, the better the mental health was. Additionally, residents in regions with greater location advantages had better mental health.

In Model 7, air pollution perception (β = −0.06, *p* < 0.001) was added. The more severe the air pollution perceived by residents was, the worse their mental health was, thereby supporting Hypothesis 4.

**Figure 1:**
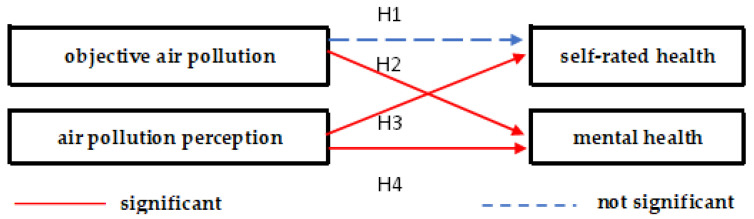
Objective air pollution, air pollution perception, and health.

Based on the comprehensive models mentioned above, it can be concluded that air pollution perception had a significant negative impact on residents’ self-rated health and mental health, while objective air pollution only had a significant negative impact on mental health (Figure 1). This study’s findings are consistent with Kim et al.’s [83] findings. Residents’ socioeconomic status, especially education and social class, had a significant positive impact on self-rated health and mental health, which confirms the findings of Yang et al. [84] and Ou et al. [73]. One possible explanation is that people with higher education levels have stronger environmental and health awareness, have higher health literacy, and are more capable of avoiding and protecting themselves from air pollution. People with a higher social class are more likely to convert their income into health benefits that can alleviate the impact of air pollution on health. Residents’ health needs, mainly in terms of daily activity capacity, also had a significant positive impact on health. In contrast to Blocker et al.’s [85] findings, this study did not find any gender differences. This study also found that per capita GDP (β = 0.02, *p* > 0.05 in Model 3; β = 0.00, *p* > 0.05 in Model 7) did not have a significant impact on health, which is inconsistent with the findings of Geng et al. [86] The reason for this discrepancy could be that this study focused on residents’ self-rated health and mental health, while Geng et al. studied population mortality rates, which might lead to different results due to differences in the dependent variables used.

### 4.2. Robustness Analysis

To further confirm the impact of objective and subjective indicators of air pollution on residents’ health, this study conducted robustness tests using other measures of pollution levels and residents’ health. We selected per capita smoke and dust particle emission as an objective indicator of air pollution instead of SO_2_. Model 4 in Table 2 is a multilevel model that used the same regression method as Model 3. Model 8 in Table 3 is a multiple linear regression model that used the same regression method as Model 7. As shown in Model 4, per capita smoke and dust particle emission (β = 7.44, *p* > 0.05) were not significantly related to self-rated health; thus, Hypothesis 1 is not supported. However, as shown in Model 8, per capita smoke and dust particle emission (β = −6.67, *p* < 0.05) were significantly related to mental health, therefore supporting Hypothesis 2. The coefficient of air pollution perception (β = −0.04, *p* < 0.001 in Model 4; β = −0.06, *p* < 0.001 in Model 8) was significantly negative, indicating a significant negative impact of air pollution perception on residents’ self-rated and mental health, thereby supporting Hypotheses 3 and 4. The test results for all four hypotheses remain unchanged.

In addition, this study reassigned the five categories of self-rated health and mental health into binary variables, with 1–2 being assigned as 0 to indicate poor health and 3–5 being assigned as 1 to indicate good health. The ICC was 0.057 for self-rated health and 0.021 for mental health, using multiple linear regression. The estimation results are not explained in detail in this paper. The results show that the objective indicator of air pollution had no significant (β = 0.74, *p* > 0.05) impact on residents’ self-rated health but did significantly (β = −2.45, *p* < 0.05) affect mental health. Air pollution perception had a significant negative impact on residents’ self-rated and mental health at the 5% (β = −0.01, *p* < 0.05) and 1% (β = −0.01, *p* < 0.01) significance levels. The above analysis confirms the robustness of the findings on the impact of objective and subjective indicators of air pollution on residents’ health across various econometric model specifications.

## 5. Discussion

In recent years, China has been promoting two initiatives, the Healthy China and Beautiful China initiatives, which are concrete manifestations aiming to improve people’s well-being and enhancing their quality of life. The Beautiful China initiative can greatly promote the Healthy China initiative by providing fresher air to breathe, cleaner water to drink, and safer soil for crop production, all of which can reduce the public’s burden of disease and improve their health from different perspectives. Using the CGSS2017 data as well as matched province-level socioeconomic indicators and air pollution data, this study found that improving the public’s subjective perception of environmental pollution is crucial, as it affects residents’ evaluation of their health. Furthermore, objective pollution also has a significant negative impact on residents’ mental health.

First, air pollution is a highly complex issue that requires more consideration of the public’s subjective perception of it. In China, air pollution has been a prominent public issue in the past decade. In official discourse and public discourse, air pollution has always been viewed as an objective social fact. However, air pollution has only become a social problem in recent years, with people subjectively perceiving it as a problem and hoping to take concerted action to solve it. We should not only focus on air pollution as an established social fact but also consider the physical and mental impacts it has on individuals as a social problem. This study found that people’s subjective perception of environmental pollution is very important and has a direct impact on health evaluation. Therefore, the government’s environmental governance work needs to include surveys to collect data on public subjective perception. Previous studies have suggested that to gain a comprehensive understanding of urban environmental governance, subjective perception and objective indicators should be used in combination. It is important and crucial for policymakers to recognize this. In recent years, the role of environmental psychology in the formulation of environmental protection policies has gradually received attention and recognition. For example, China’s 13th Five-Year Plan emphasized that the formulation and implementation of environmental protection policies should be coordinated with the public’s intuitive perception. This indicates that environmental psychology has great potential in evaluating the feasibility of environmental protection policies and helping to solve environmental problems. Taking air pollution as an example, on the one hand, the public usually judges air quality based on sensory experiences, such as visibility and odor, while objective air quality indicators are based on physical indicators of damage to human health. Subjective and objective indicators of air pollution often do not match, but the public’s subjective perception can be used to optimize objective air quality indicators. In fact, as early as the 1980s, scholars improved the air quality index value by adjusting the public’s threshold for sensing air pollution [87], which is worth learning from. On the other hand, the harm of air pollution to the physical and mental health of the public not only depends on the degree of direct exposure to pollution but also on the public’s subjective evaluation of air pollution. From the perspective of air pollution perception, exploring how to help the public cope with the harm of air pollution can be approached from two paths: physical (such as wearing masks and reducing outdoor activities) and psychological (such as rational evaluation of air pollution). The Chinese government should incorporate public perception of air pollution into the air quality index system to respond to the non-convergence of public perception and detection results and to better carry out pollution control.

Secondly, health should be a measure of environmental justice. Human health and survival require a good and safe spatial environment. Basic human rights, such as the rights to life, health, property, and happiness, can only be realized in a suitable living space. In this sense, eliminating spatial injustice and maintaining individual environmental justice should become part of the protection of basic human rights. The 1972 United Nations Declaration of the Human Environment stated that humans have the right to live in an environment that enables them to live with dignity and well-being, with the basic rights to freedom, equality, and adequate living conditions. It is our solemn responsibility to protect and improve the environment for current and future generations. Today, people generally recognize that the right to life and health is the most basic human right, and a good ecological environment is the guarantee of human life and health, as well as the basic conditions for human survival, development, and dignified life. Human environmental health problems are usually divided into two categories: (1) traditional environmental health problems related to poverty and underdevelopment, such as lack of safe and clean drinking water and health damage caused by natural disasters, and (2) modern environmental health problems related to industrial production and lifestyle, such as air pollution caused by petrochemical industries and modern agriculture and infectious diseases repeatedly occurring due to dense urban populations. Currently, environmental health problems and related health risks are beginning to shift from the traditional to modern type, and health damage caused by deteriorating spatial environments is constantly emerging. Therefore, a good living space and a good living environment are the direct guarantee of life and health and the basic conditions for people to enjoy a happy life. In view of this, life and health in spatial environments should be regarded as a measure of environmental justice. Human living and residential spaces obviously belong to the artificial environment constructed by society and are heterogeneous due to the significant differences in living spaces among different classes and social groups, which are related to different living standards and health conditions. Zivin et al. [88] and Deschenes et al. [89] pointed out that there are differences in cognitive abilities and behaviors among people with different educational levels, and these behaviors are important factors determining the health effects of air pollution. This study also found that people with higher socioeconomic status have better self-rated health and mental health. We need to strive to maintain socio–spatial harmony and eliminate discrimination and prejudice in the pursuit of spatial environmental justice. The Chinese government should introduce relevant supportive policies in a timely manner to prevent the unfairness of air pollution from expanding health inequality.

## 6. Conclusions

Air pollution has become a common challenge faced by humanity, causing damage to public health. This paper presented empirical evidence from China, using data from the CGSS2017 and matched province-level socioeconomic indicators and air pollution data. Regression analysis was performed to analyze the relationship between objective indicators of air pollution, subjective perception of air pollution severity, and public health assessment. This paper presented four hypotheses. H1: The higher the objective air pollution level, the worse the self-rated health will be. H2: The higher the objective air pollution level, the worse the mental health will be. H3: The higher the air pollution perception, the worse the self-rated health will be. H4: The higher the air pollution perception, the worse the mental health will be. We concluded that H1 is not supported, while H2–4 are supported by the data. One possible explanation is that, in China, air pollution is constructed as a well-known social problem, and air pollution perception has a significant impact on residents’ health. Furthermore, the study used self-reported health status, which is more closely related to perception rather than objective air pollution. In our study, no statistical significance was observed between air quality and self-rated health. Self-rated health is a subjective measure of health status and may have some deviation from objective health measures. There may be limitations to the data, so caution is needed when interpreting the results of this study.

The study findings reveal that objective air pollution has a significant negative impact on mental health. Subjective perception of air pollution has a significant negative impact on self-rated health and mental health, suggesting that it needs more consideration to reduce environmental health risks. Therefore, the problem of air pollution urgently needs to be addressed. Firstly, the Chinese government needs to effectively improve its objective performance of environmental governance, enhance people’s well-being, and strengthen its investigation and data collection with regard to public perception. Secondly, pollution control can be an effective method of promoting health and hygiene. Environmental governance should focus on solving prominent environmental problems that harm public health, as well as strengthening spatial environmental justice considerations in the dimensions of life and health. We also found evidence of health inequality related to socioeconomic status indicators. Future research can explore the issue of inequality regarding the health effects of air pollution on different social groups. This belongs to the research field of health inequality. Currently, this field focuses more on physiological health inequality and the distribution of some traditional psychological diseases (such as depression and schizophrenia) and related inequality issues in different populations. There have been some social psychology studies on air pollution and health inequality, but further interdisciplinary research is needed from different fields, such as medicine, psychology, sociology, and public management, to explore the unequal effects of air pollution.

## Figures and Tables

**Table 1:** Descriptive statistics of variables.

Variable	Obs	M	SD	Minimum	Maximum	Level
Self-rated health	4132	3.48	1.09	1	5	individual
Mental health	4132	3.80	1.00	1	5	individual
Gender	4132	0.54	0.50	0	1	individual
Age	4132	49.95	16.92	17	95	individual
Years of education	4132	9.06	4.64	0	19	individual
Income	4132	7.70	5.41	−4.61	16.11	individual
Hukou	4132	0.64	0.48	0	1	individual
Political status	4132	0.11	0.32	0	1	individual
Self-assessed class	4132	4.14	1.68	1	10	individual
Marital status	4132	0.76	0.43	0	1	individual
Family size	4132	2.83	2.05	1	12	individual
Region	4132	2.22	0.79	1	3	individual
Daily activity ability	4132	3.91	1.14	1	5	individual
Pollution perception	4132	2.91	1.21	1	6	individual
GDP per capita	28	6.54	3.02	2.76	11.82	province
GDP growth rate	28	0.07	0.02	−0.03	0.11	province
Internet penetration rate	28	0.56	0.12	0.40	0.78	province
SO_2_ emissions per capita	28	0.01	0.01	0.00	0.04	province
Smoke and dust particle emissions per capita	28	0.01	0.01	0.00	0.03	province

Note: natural pairs of income were used.

**Table 2:** Factors influencing self-rated health.

Variable	Model (1)	Model (2)	Model (3)	Model (4)
Gender		−0.02	−0.02	−0.02
		(0.03)	(0.03)	(0.03)
Age		−0.01 ***	−0.01 ***	−0.01 ***
		(0.00)	(0.00)	(0.00)
Years of education		0.02 ***	0.02 ***	0.02 ***
		(0.00)	(0.00)	(0.00)
Income		0.01 *	0.01 *	0.01 *
		(0.00)	(0.00)	(0.00)
Hukou		0.09 **	0.09 **	0.09 **
		(0.03)	(0.03)	(0.03)
Political status		0.04	0.03	0.03
		(0.04)	(0.04)	(0.04)
Self-assessed class		0.08 ***	0.07 ***	0.07 ***
		(0.01)	(0.01)	(0.01)
Marital status		−0.05	−0.06	−0.06
		(0.03)	(0.03)	(0.03)
Family size		0.01	0.01	0.01
		(0.01)	(0.01)	(0.01)
Region		0.10	0.10	0.11 *
		(0.06)	(0.06)	(0.05)
Daily activity ability		0.48 ***	0.48 ***	0.48 ***
		(0.01)	(0.01)	(0.01)
GDP per capita		0.02	0.02	0.02
		(0.02)	(0.02)	(0.02)
GDP growth rate		−0.72	−0.59	0.06
		(1.60)	(1.59)	(1.60)
Internet penetration rate		−1.04	−1.06	−1.12 *
		(0.58)	(0.58)	(0.56)
SO_2_ emissions per capita		2.54	2.83	
		(5.12)	(5.11)	
Pollution perception			−0.04 ***	−0.04 ***
			(0.01)	(0.01)
Smoke and dust particle emissions per capita				7.45
				(5.01)
Constant	3.45 ***	2.00 ***	2.12 ***	2.04 ***
	(0.06)	(0.31)	(0.31)	(0.30)
Provincial-level variance	0.08			
Individual-level variance	1.13			
N	4132	4132	4132	4132

Note: the numbers in parentheses are standard errors. * *p* < 0.05, ** *p* < 0.01, *** *p* < 0.001.

**Table 3:** Factors influencing mental health.

Variable	Model (5)	Model (6)	Model (7)	Model (8)
Gender		−0.05	−0.05	−0.05
		(0.03)	(0.03)	(0.03)
Age		0.01 ***	0.01 ***	0.01 ***
		(0.00)	(0.00)	(0.00)
Years of education		0.01 **	0.01 **	0.01 **
		(0.00)	(0.00)	(0.00)
Income		−0.00	0.00	0.00
		(0.00)	(0.00)	(0.00)
Hukou		0.03	0.04	0.03
		(0.03)	(0.03)	(0.03)
Political status		0.07	0.06	0.06
		(0.05)	(0.05)	(0.05)
Self-assessed class		0.06 ***	0.06 ***	0.06 ***
		(0.01)	(0.01)	(0.01)
Marital status		0.08 *	0.07 *	0.07 *
		(0.03)	(0.03)	(0.03)
Family size		−0.00	−0.01	−0.01
		(0.01)	(0.01)	(0.01)
Region		0.07 *	0.08 **	0.09 **
		(0.03)	(0.03)	(0.03)
Daily activity ability		0.38 ***	0.38 ***	0.38 ***
		(0.01)	(0.01)	(0.01)
GDP per capita		0.00	0.00	0.00
		(0.01)	(0.01)	(0.01)
GDP growth rate		−0.66	−0.45	−0.70
		(0.81)	(0.81)	(0.86)
Internet penetration rate		−0.08	−0.08	−0.07
		(0.28)	(0.28)	(0.28)
SO_2_ emissions per capita		−8.17 **	−7.86 *	
		(3.06)	(3.05)	
Pollution perception			−0.06 ***	−0.06 ***
			(0.01)	(0.01)
Smoke and dust particle emissions per capita				−6.67 *
				(3.27)
Constant	3.73 ***	1.58 ***	1.76 ***	1.73 ***
	(0.05)	(0.17)	(0.18)	(0.18)
Provincial-level variance	0.05			
Individual-level variance	0.94			
N	4132	4132	4132	4132

Note: the numbers in parentheses are standard errors. * *p* < 0.05, ** *p* < 0.01, *** *p* < 0.001.

## Data Availability

The dataset used in this study can be obtained from the corresponding author on reasonable request.
